# Disruption of BASIGIN decreases lactic acid export and sensitizes non-small cell lung cancer to biguanides independently of the LKB1 status

**DOI:** 10.18632/oncotarget.2862

**Published:** 2014-12-03

**Authors:** Sara Granja, Ibtissam Marchiq, Renaud Le Floch, Conceição Souto Moura, Fátima Baltazar, Jacques Pouysségur

**Affiliations:** ^1^ Life and Health Sciences Research Institute (ICVS), School of Health Sciences, University of Minho, Braga, Portugal; ^2^ ICVS/3B's-PT Government Associate Laboratory, Braga/ Guimarães, Portugal; ^3^ Institute for Research on Cancer and Aging of Nice (IRCAN), Centre A. Lacassagne, Nice, France; ^4^ Centre Scientifique de Monaco (CSM), Monaco; ^5^ Department of Pathology, Centro Hospitalar de São João, Porto, Portugal; ^6^ Institute of Molecular Pathology and Immunology of the University of Porto (IPATIMUP) and Medical Faculty of University of Porto, Porto, Portugal

**Keywords:** lung cancer, CD147, BASIGIN, monocarboxylate transporters, MCTs, lactate, glycolytic metabolism, metformin, ZFNs

## Abstract

Most cancers rely on aerobic glycolysis to generate energy and metabolic intermediates. To maintain a high glycolytic rate, cells must efficiently export lactic acid through the proton-coupled monocarboxylate transporters (MCT1/4). These transporters require a chaperone, CD147/BASIGIN (BSG) for trafficking to the plasma membrane and function.

To validate the key role of these transporters in lung cancer, we first analysed the expression of MCT1/4 and BSG in 50 non-small lung cancer (NSCLC) cases. These proteins were specifically upregulated in tumour tissues. We then disrupted BSG in three NSCLC cell lines (A549, H1975 and H292) *via* ‘Zinc-Finger Nucleases’. The three homozygous *BSG^−/−^* cell lines displayed a low MCT activity (10- to 5-fold reduction, for MCT1 and MCT4, respectively) compared to wild-type cells. Consequently, the rate of glycolysis, compared to the wild-type counterpart, was reduced by 2.0- to 3.5-fold, whereas the rate of respiration was stimulated in *BSG^−/−^* cell lines. Both wild-type and BSG-null cells were extremely sensitive to the mitochondria inhibitor metformin/phenformin in normoxia. However, only BSG-null cells, independently of their LKB1 *status*, remained sensitive to biguanides in hypoxia *in vitro* and tumour growth in nude mice. Our results demonstrate that inhibiting glycolysis by targeting lactic acid export sensitizes NSCLC to phenformin.

## INTRODUCTION

Non-small cell lung cancer (NSCLC) is one of the most common causes of death from cancer worldwide, accounting in 2009 for 30% and 26% of all male and female cancer deaths, respectively [34]. The overall 5-year survival rate of patients with metastatic disease remains less than 15% [7]. Thus, a better understanding of the molecular mechanisms driving lung tumourigenesis is crucial and will provide enormous benefit in developing new pharmacological treatments.

Tumour cells are characterized by excessive aerobic glycolysis that is a hallmark of cancer cells [1, 31]. The high consumption of glucose and consequent high production of lactate is a constant feature of most cancers, therefore high expression of glucose transporters and glycolytic enzymes is widely observed [2, 3, 8, 14, 24]. In addition, it is necessary to extrude the lactate produced to avoid lactic acidosis, which may act as a negative feedback mechanism to inhibit glycolysis [24, 35]. Monocarboxylate transporters (MCTs) constitute a family of 14 proton-linked plasma membrane transporters. Only MCT1 and MCT4 have been consistently shown to be upregulated in several human cancers [25] and to play an important role in maintaining glycolysis. For localization in the plasma membrane and for activity, these isoforms require a chaperone named CD147/BASIGIN (BSG) [15, 30, 38]

BSG, also known as EMMPRIN (Extracellular Matrix Metalloproteinase Inducer), is an evolutionary conserved protein that belongs to the immunoglobulin (Ig) superfamily. It is a widely distributed heavily glycosylated type I transmembrane glycoprotein [14, 15] and is expressed at high levels mainly in metabolically active cells such as lymphoblasts and malignant tumour cells [9, 37, 40]. BSG has been proposed to effect metalloproteinases (MMP) that degrade the extracellular matrix [37], tumour growth, chemoresistance [12, 13, 41, 42] and metastasis [22, 37]. However, the role of MCTs/BSG in lung cancer is controversial and poorly elucidated [16, 20, 27]. Therefore, we aimed to understand the role of MCTs/BSG in lactic acid extrusion, pH homeostasis and lung tumour growth. In this study we first observed that MCT1, MCT4 and BSG were overexpressed in human lung cancers when compared to adjacent non-tumour tissue. Therefore, we chose three NSCLC cell lines (A549, H1975 and H292) one of which was defective in LKB1 (A549) to analyse the impact of targeting the MCT activity on energy metabolism, on *in vitro* cell proliferation, and on *in vivo* tumour growth. We exploited RNA interference knockdown or Zinc Finger Nucleases knockout of a single gene, *BSG,* to reduce the activity of both MCT1 and MCT4. We report that the disruption of *BSG* decreased the expression and activity of MCT1 and MCT4, decreased the rate of glycolysis, increased the rate of respiration and sensitized the three *BSG^−/−^* tumour cell lines to the inhibition of OXPHOS by metformin/phenformin (mitochondrial complex I inhibitors) *in vitro* and *in vivo*.

## RESULTS

### MCT expression in lung tumours

**Figure 1:**
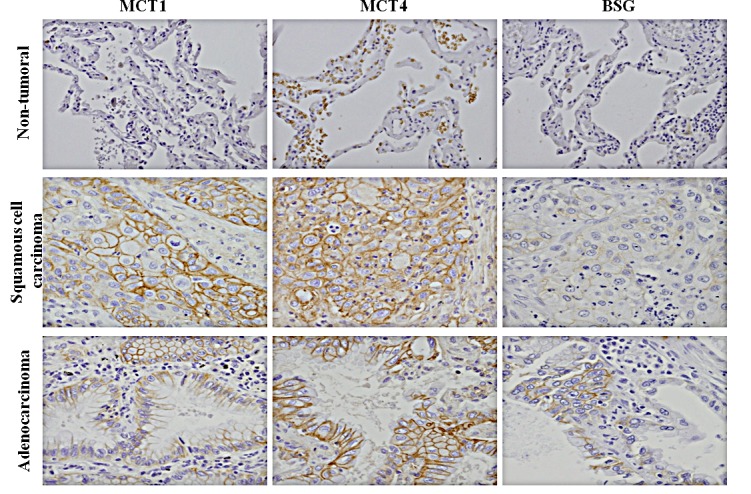
Immunohistochemical expression of the monocarboxylate transporters, MCT1 and MCT4, and their chaperone protein BSG in lung cancer samples All markers were upregulated at the plasma membrane of tumour cells. Pictures were obtained using the microscope Olympus BX61, at 40 magnification.

First, we analysed the expression of MCTs and BSG in a series of human lung cancer tissues. We studied 50 cases of non-small cell lung cancer (NSCLC). The series comprised squamous cell carcinoma tumours (n=8), adenocarcinomas (n=29) and other types (n=13). All the cases had available adjacent non-tumour tissue that was also studied. We observed that MCT1, MCT4 and BSG were significantly overexpressed in tumour samples when compared to adjacent non-tumour tissue (Fig. 1, Table 1). According to the IARC, the most common histological types are squamous cell carcinomas and adenocarcinomas [36]. The levels of the three components of the lactate transport complex differed between the two histological types. MCT1 and MCT4 were expressed in all squamous cell carcinoma type cases, and BSG was expressed in 60% of the cases. MCT1, MCT4 and BSG were expressed in 20%, 30% and 40% of the cases of adenocarcinomas, respectively (Table 1).

**Table 1:** Expression of monocarboxylate transporters (MCT1 and MCT4) and BSG in non-tumour and lung tumour tissues

Isoform	n	Plasma membrane expression	
		Positive(%)	*p*
**MCT1**			
Nontumoral	50	0(0%)	
SCC	8	8(100%)	<.001
AD	29	9(31%)	
Other types	13	1(7.7%)	
**MCT4**			
Nontumoral	50	0(0%)	
SCC	8	8(100%)	<.001
AD	29	15(51.7%)	
Other types	13	2(15.4%)	
**BSG**			
Nontumoral	50	0(0%)	
SCC	8	5(62.5%)	<.001
AD	29	16(55.2%)	
Other types	13	2(15.4%)	

SCC: Squamous cell carcinoma, AD: Adenocarcinoma;

*p* association between non-tumour and tumour samples

### Downregulation of BSG and MCT4 sensitizes A549 cells to oligomycin in normoxia

**Figure 2:**
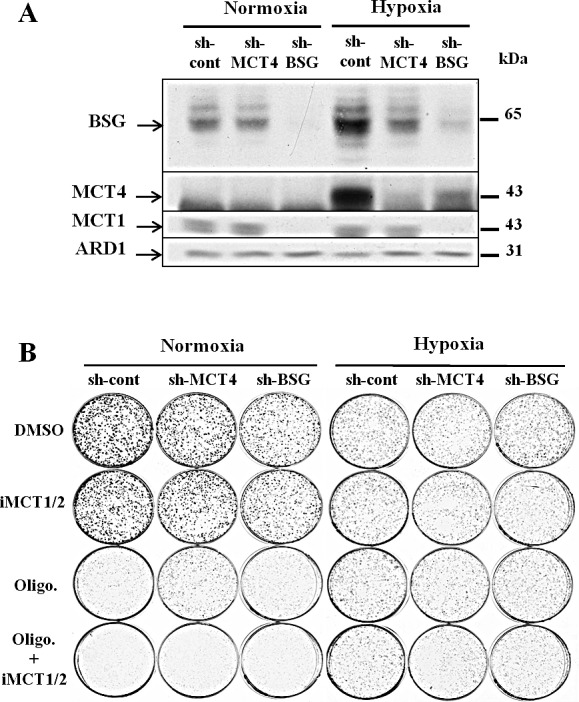
Downregulation of MCT4 and BSG A: Immunoblot analysis of MCT1, MCT4 and BSG in cells transfected with either scrambled shRNA or shRNA targeting MCT4 and BSG and culture in normoxia (21% O_2_) and hypoxia (1% O_2_). ARD1 used as a loading control; B: Clonal growth in the absence or presence of oligomycin (1μg/mL) or iMCT1/2 AR-C155858 (300nM) or both compounds either in normoxia and hypoxia for 8 days.

After confirming the expression of these biomarkers in lung tumours and taking into account the overlapping activity of both MCT1 and MCT4, we decided to evaluate the effect on growth of MCT4 and BSG silencing in A549 cells or of MCT1 pharmacological inhibition (AstraZeneca, iMCT1/2; AR-C155858). The silencing of MCT4, by shRNA, decreased MCT4 and BSG expression in hypoxia (Fig. 2A). Moreover, as expected, BSG silencing induced a parallel decrease in the expression of MCT1 and MCT4 in both normoxia and hypoxia (Fig. 2A). Silencing of MCT4 or BSG had only very modest effect on clonal growth in normoxia and hypoxia even in the presence of iMCT1/2 (Fig. 2B). Blockade of OXPHOS by oligomycin did not impact on the growth rate when the cells were cultured in hypoxia (1% O_2_) (Fig. 2B, right panel). By contrast, cell growth was severely affected in normoxia by oligomycin, which was magnified in the presence of iMCT1/2 (Fig. 2B). This experiment demonstrated that hypoxic cells, in spite of appreciable silencing of MCT4 and MCT1 inhibition, remained resilient to growth inhibition by targeting glycolysis. Indeed, shRNA targeting of MCT4 did not totally abolish the activity of this transporter, and the residual expression might explain this hypoxic resistance to MCT1 and OXPHOS blockade. In the absence of a specific pharmacological inhibitor of MCT4, and taking into account the interdependency between MCTs and their chaperone, we decided to develop BSG-null cells to further explore the role of MCTs in targeting glycolysis and tumour growth.

### Generation of *BSG* ^−/−^ lung cancer cell lines using Zinc Finger Nucleases

**Figure 3:**
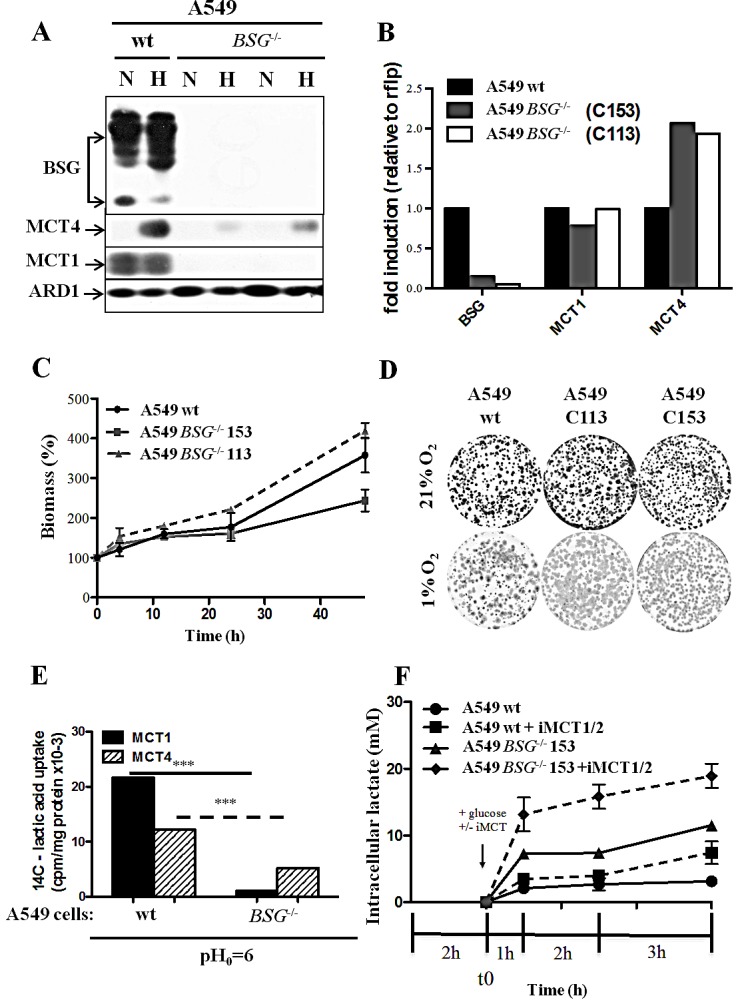
Effect of *BSG* disruption in lung carcinoma cell line A549 A: Immunoblot analysis of MCT1, MCT4 and BSG in wt and *BSG*^−/−^ A549 cell lines maintained in normoxia (N) or hypoxia (1% O_2_) (H) for 48h. ARD1 was used as a loading control; B: mRNA levels of MCT1, MCT4 and BSG in wt and *BSG*^−/−^ cells (Clones C153 and C113) in hypoxia 1% O_2_ C: Total biomass of wt and *BSG*^−/−^ A549 (Clones C153 and C113) cells was evaluated over time using the sulphorhodamine B assay; D: Clonal growth of wt and *BSG*^−/−^ cells (Clones C153 and C113) in normoxia (21% O_2_) and hypoxia (1% O_2_); E: [^14^C]-Lactic acid uptake of wt and *BSG*^−/−^ A549 cells in the absence or presence of iMCT1/2 (300nM) in hypoxia (1% O_2_); F: Time-course of intracellular lactate concentration in response to glucose (25mM) added in the presence of either DMSO or iMCT1/2 (300nM). (*** p <0.0001).

**Figure 4:**
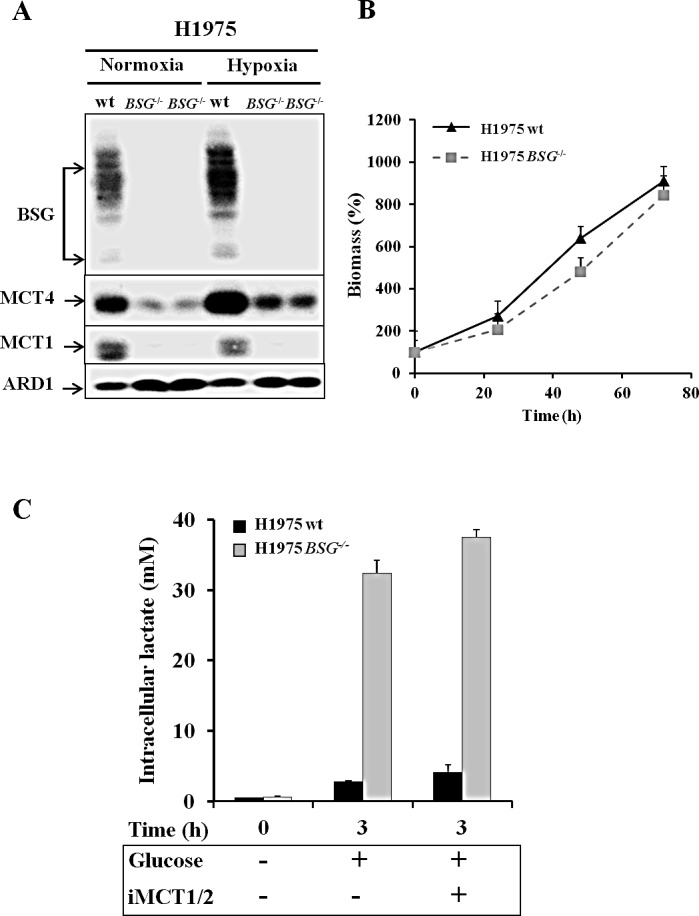
Effect of *BSG* disruption in lung carcinoma cell line H1975 A: Immunoblot analysis for MCT1, MCT4 and BSG in wt and *BSG*^−/−^ H1975 cell lines maintained in normoxia or hypoxia (1% O_2_) for 48h. ARD1 was used as a loading control; B: Total biomass of wt and *BSG*^−/−^ H1975 cells was evaluated over time using the sulphorhodamine B assay; C: Time-course of the intracellular lactate concentration in response to glucose (25mM) added in the presence of either DMSO or iMCT1/2 (300nM).

First, we disrupted the *BASIGIN* (*BSG*) gene in three different non-small lung cancer cell lines (A549, H1975 and H292) by using Zinc Finger Nucleases. Knocked-out candidates lacking expression of BSG (Figs. 3A, 4A) were identified and selected for further characterization. The site that was cut was amplified by PCR and the sequence and mutational independent status of the clones was confirmed by sequencing. Next, we analysed the expression of the MCT1 and MCT4 isoforms in normoxia and hypoxia 1% O_2_ in these cells Immunoblotting showed that the MCT1 protein was not detectable in *BSG^−/−^* A549 cells compared to wild-type (wt) cells, while MCT4 expression was highly reduced. We also observed that the non-detectable MCT4 expression in normoxia in *BSG^−/−^* cells remained inducible in hypoxia (Fig. 3A). Similar results were obtained for the H1975 (Fig. 4A) and H292 cell lines (data not shown). Knockout (KO) of the *BSG* gene in these cells induced a huge decrease in the protein level of MCT1 and MCT4 in both normoxia and hypoxia.

Moreover, we demonstrated by qPCR a low level of transcript expression of BSG, but no evidence of changes in mRNA expression of MCT1, while MCT4 expression increased by 2-fold in both A549 *BSG^−/−^* clones (Fig. 3B). This stability or even increase in MCT4 mRNA contrasted with the virtual loss of the corresponding gene product. We further assessed the impact of *BSG* disruption on the *in vitro* growth rates of the A549- (Fig. 3C, 3D) and H1975-derived cell lines (Fig. 4B) in normoxia. All BSG-null cells, excepting the *BSG^−/−^* A549 clone (C153), displayed a normal growth rate compare to the parental cells (reduction of less than 5% for *BSG^−/−^* cells).

### *BSG* gene disruption inhibits the MCT activity and increases the intracellular lactate pool

We quantified the membrane activity of both MCT1 and MCT4 by a direct measure of the initial rates of lactic acid transport in hypoxic A549 parental and *BSG^−/−^* cells. The uptake of ^14^C-lactic acid was measured at a low external pH (6.0) to favour its entry in the absence or presence of the specific iMCT1/2 (Fig. 3E). Thus, we demonstrated that the ability of MCT1 to transport lactate was severely suppressed (>90%) in *BSG^−/−^* cells, while that of the hypoxia-induced MCT4 is only decreased by 60% compared to parental cells. The transport activity of MCT1/4 was also evaluated by measuring the intracellular lactic acid pools (Fig. 3F). After 1h of glucose starvation, A549-derived cells rapidly accumulated intracellular lactic acid on addition of glucose to reach an equilibrium state within 3h. In accordance with the above experiments on the MCT transport activity, *BSG^−/−^* A549 cells accumulated a much higher level of intracellular lactic acid than parental cells. From 2-3mM lactate in parental cells to 11-12mM in *BSG^−/−^* cells (Fig. 3F). Treatment of the cells with iMCT1/2 further increased the intracellular lactate pool of the *BSG*-null cells (20mM) whereas parental cells remained less sensitive.

In the highly glycolytic H1975 cell line, our results showed that the *BSG* disrupted cells accumulated higher levels of intracellular lactic acid (33mM), than the wild-type cells (3mM) (Fig. 4C) and the *BSG^−/−^* A549 cells (Fig. 3F). However, these cells remained less sensitive to MCT1 inhibition due to the high decrease in MCT1 expression of *BSG* KO (Fig. 4A).

Taken together, these findings demonstrate that BSG plays a central role in controlling lactic acid export and therefore the rate of glycolysis of non-small lung cancer cells.

### Disruption of *BSG* decreases the rate of glycolysis and increases OXPHOS in different lung cancer cell lines

**Figure 5:**
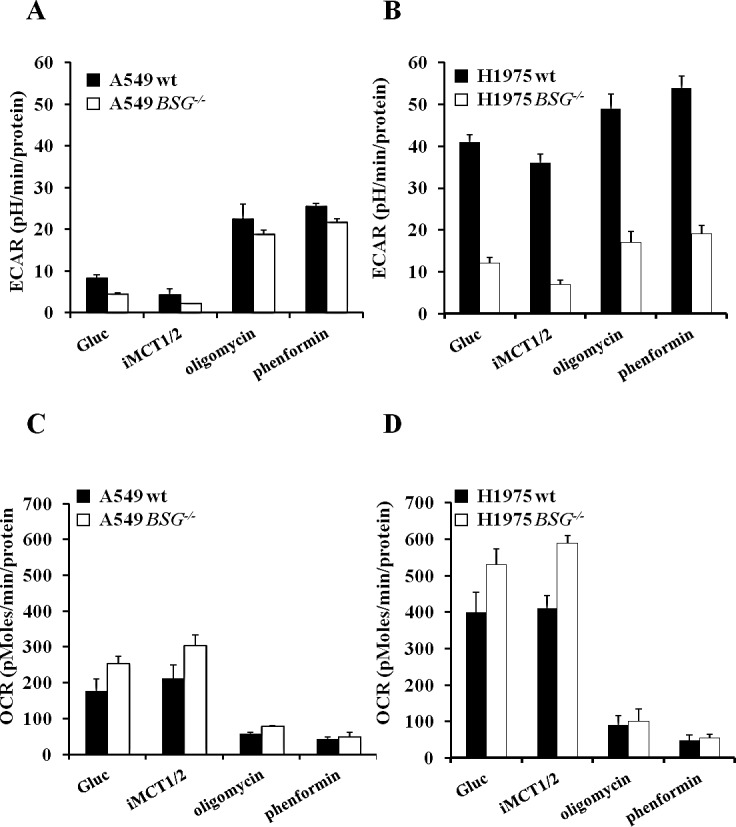
Analysis of bioenergetic pathways in lung carcinoma cells disrupted for *BSG* A, B: Real time analysis of the extracellular acidification rate (ECAR) of wt and *BSG*^−/−^ A549 (A) or H1975 (B) cells using a Seahorse XF24 analyzer after injection of glucose (10mM), iMCT1/2 (1μM), oligomycin (1μg/mL) or phenformin (50μM) ; C, D: Real time analysis of the oxygen consumption rate (OCR) of wt and *BSG*^−/−^ cells A549 (C) or H1975 (D) using a Seahorse XF24 analyzer after injection of the compounds described above. Data represent the average of at least three independent experiments.

To further investigate the interplay between glycolysis and OXPHOS, under the conditions where lactic acid export was pharmacologically or genetically reduced, we used the Seahorse XF24 Analyser to measure the extracellular acidification rate (ECAR) and oxygen consumption rate (OCR) of our wt and *BSG^−/−^* cell lines. In the presence of glutamine (2mM) and glucose (10mM), BSG-null A549 cells showed a 2-fold reduction in ECAR compared to wt cells (Fig. 5A), whereas the more glycolytic cell line H1975 (ECAR=40 in H1975 and 10 in A549) showed a 3.4-fold reduction in ECAR upon *BSG* KO compared to parental cells (Fig. 5B). EACR values were further reduced (1.8-fold) in A549- and H1975-derived cell lines in the presence of iMCT1/2, reflecting inhibition of glycolysis by limiting lactic acid export (Fig. 5A, B). Addition of oligomycin or phenformin stimulated by 3- to 4-fold ECAR, respectively in wt and *BSG^−/−^* A549 cells. However, the ECAR of wt and *BSG^−/−^* H1975 cells was only increased by 1.3- to 1.4-fold, respectively (Fig. 5A, B). These results indicate that, in contrast to the H1975 cell line, A549 cells were moderately glycolytic as they only used one third of their glycolytic potential.

By contrast, the OCR values were high in both A549 and H1975 cell lines. These values were even increased in BSG-null cells compared to wt cells and further increased in response to MCT1 inhibition (Fig. 5C, D). Respiration was dramatically reduced in response to the mitochondrial complex I inhibitor phenformin (Fig. 5C, D).

### MCT inhibition sensitizes lung cancer cells to phenformin in hypoxia

**Figure 6:**
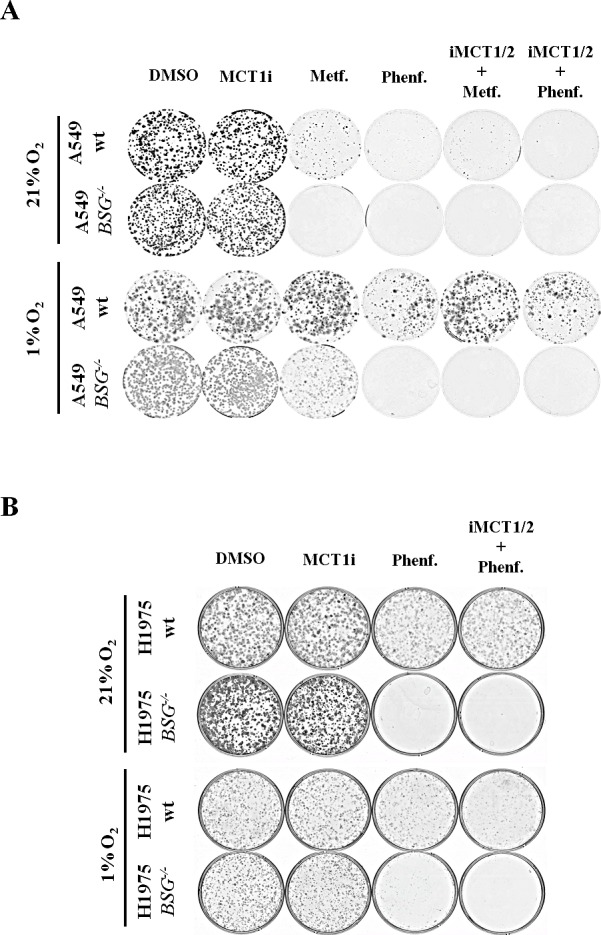
Disruption of *BSG* sensitizes lung carcinoma cells to phenformin in both normoxia and hypoxia A: Clonal growth of wt and *BSG*^−/−^ A549 cells in the presence or absence of iMCT1/2 (300nM), metformin (Metf.-1mM), phenformin (Phenf. -50μM) or in combination. Cells were maintained in normoxia for 10 days (upper panel) and in hypoxia (1% O_2_) for 15 days (lower panel); B: Clonal growth of wt and *BSG*^−/−^ H1975cells in the presence or absence of iMCT1/2 (300nM), Phenformin (Phenf. -50μM) or in combination iMCT1/2. Cells were maintained in normoxia for 10 days (upper panel) and in hypoxia (1% O_2_) for 12 days (lower panel).

Deletion of *BSG* did not affect *in vitro* clonal growth of either A549 or H1975 cells, even in the presence of iMCT1/2 (Fig. 6A, B). However, only wt A549 cells displayed an extremely high sensitivity to metformin/phenformin or oligomycin (not shown), while the more glycolytic H1975 cells remain insensitive (Fig. 6B). This is in agreement with the high oxidative/low glycolytic activity of A549 cells and perhaps also with the loss of LKB1 (see discussion) (Fig. 6A). However, in hypoxia, parental A549 cells lost sensitivity to OXPHOS inhibitors, which is consistent with optimal induction of glycolytic enzymes through stabilization of the hypoxia-inducible factor (HIF). In sharp contrast to wt cells, BSG-null cells A549 and H1975 retained a high level of sensitivity to phenformin/metformin alone or in combination with iMCT1/2 (Fig. 6A, B). This striking difference between wt and BSG-null cells in hypoxia could easily be explained by the failure of *BSG^−/−^* cells to express sufficient hypoxia-inducible MCT4 to sustain glycolysis (Figs. 3A, 4A).

These results were confirmed in another glycolytic non-small lung carcinoma cell line, H292, disrupted for the *BSG* gene (data not shown), highlighting the fact that targeting lactic acid export sensitizes lung carcinoma cell lines to biguanides and thus suppresses their growth in both normoxia and hypoxia.

### Combination of inhibition of glycolysis and OXPHOS decreases tumour growth *in vivo*

**Figure 7:**
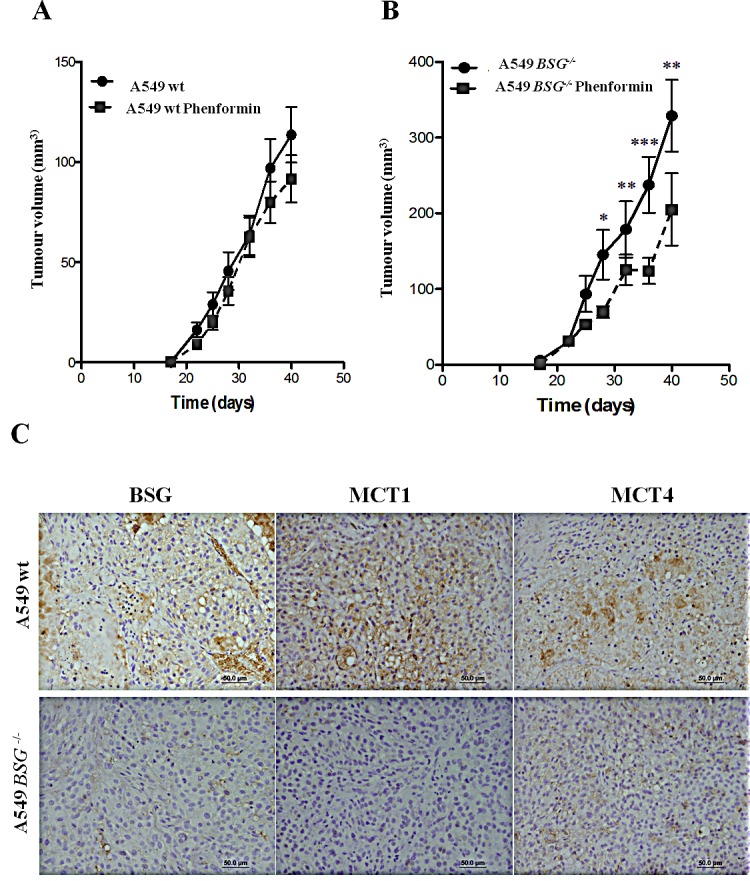
Xenograft tumour growth Wt (A) and *BSG*^−/−^ cells (B) A549 cells were s.c. injected into the back of athymic nude mice. 5% sucrose with and without phenformin (200 mg/kg) was added to the drinking water for all the mice. Ten mice were studied per condition (*p<0.05, **p<0,001, ***p<0,0001); C: Immunohistochemical expression of MCT1, MCT4 and BSG in A549 wt and A549 *BSG*^−/−^ tumor samples. Pictures were obtained using the microscope Olympus.

To assess the effect of *BSG* disruption *in vivo,* we compared the tumourigenicity of wt and *BSG^−/−^* A549 cells. Nude mice were s.c. injected with both cell populations. Surprisingly, *BSG^−/−^* cells generated bigger tumours than wt cells (Fig. 7A, B-black lines). However, phenformin greatly reduced the tumour burden in *BSG^−/−^* cells than in wt cells (Fig. 7A, B). To confirm the expression levels of BSG and MCTs, tumours were collected at the end of the experiment and tested by immunohistochemistry. As expected, BSG and MCT1 expression was not detected in *BSG*^−/−^ tumors while MCT4 was present, although with a weaker expression. Moreover, treatment with phenformin did not alter the expression of BSG, MCT1 or MCT4. (Fig. 7C).

## DISCUSSION

Upregulation of glycolysis and adaptation to acidosis are key events in the transition from *in situ* to invasive cancer [11]. MCTs may play an important role through their involvement in exporting lactic acid [10]. MCTs have been described to be upregulated in several cancers [25]. However, expression in lung cancer is still controversial [16, 20, 27]. In the present study, we showed that MCTs are upregulated in lung tumour tissue when compared to adjacent non-tumour tissue. Moreover, we detected MCT1 and MCT4 in the plasma membrane of all the histological types studied. These results were in accordance with those of Koukourakis and co-workers [16]. However, they did not describe the cellular localization and histological type of the samples stained for MCT. Interestingly, we found that MCT1 and MCT4 were overexpressed in the squamous cell carcinoma subtype and MCT4 was overexpressed in all cases of adenocarcinoma subtypes, suggesting a different expression pattern of the histological types. However, due to the small number of cases, this study should be extended and replicated to obtain more solid statistically relevant data.

To understand the biological role of MCTs, we developed a strategy to inhibit their activity in a lung carcinoma cell line. For MCT1, we used the specific MCT1/2 inhibitor (AR-C155858; AstraZeneca) and found that it did not affect *in vitro* cell growth in either normoxia or hypoxia. In normoxia, A549 cells relied mainly on OXPHOS rather than on glycolysis [39], thus inhibiting MCT1, even with low expression of MCT4, did not affect cell clonogenicity. In hypoxia, in which OXPHOS was reduced and glycolysis enhanced, cells were able to survive and proliferate due to the presence of HIF-1-induced MCT4. Although, regulation of MCT expression is still not fully understood, one of the best characterized mechanisms of MCT regulation occurs through co-expression with BSG. As no pharmacological inhibitor of MCT4 is yet available and as previous published results [17] showed that the major pro-tumour action of BSG is mediated through bioenergetics, namely by controlling lactate transport, we decided to knockdown both MCT4 and BSG. As shRNA targeting of MCT4 and BSG failed to completely stop the growth of A549 cells in hypoxia, we decided to disrupt the *BSG* gene. We observed that KO of BSG resulted in a decrease in MCT1 and MCT4 expression. We observed that the mRNA level remained unchanged for MCT1 but increased 2-fold for MCT4 in the absence of BSG. As we previously reported for the LS174 colon cell line [17], BSG KO did not affect expression of MCTs at the transcriptional/translational levels but severely reduced the trafficking of MCTs from the endoplasmic reticulum (ER) to the plasma membrane [15]. In the absence of the chaperone (BSG), MCT1 and MCT4 were very unstable in the ER, which explained their decreased expression as detected by immunoblotting (Fig. 3A) and reduced activity in lactic acid transport (Fig. 3E). Nevertheless, *BSG* gene disruption did not totally suppress the activity of MCT4 in these cells.

These results support the idea that MCTs may use an alternative chaperone for processing to the plasma membrane, as already suggested previously by our group [25, 27], or that MCTs alone could reach the plasma membrane but with a very low efficiency.

Targeting a metabolic pathway can be tricky due to the high plasticity of the metabolic networks of cancer cells. Thus, we decided to analyze the impact of disrupting BSG and OXPHOS on *in vitro* growth and tumourigenicity.

The anti-diabetic biguanide drugs metformin and phenformin used in the clinic have been shown to inhibit mitochondria complex I [6, 23], with no toxicity for metformin and little toxicity for phenformin. More recently, these biguanides have shown interesting new potential applications in oncology [5, 18, 19, 29, 32]. In this context, we report here that the three NSCLC parental cell lines (A549, H292, H1975) or their *BSG^−/−^*derivatives were highly sensitive to metformin or phenformin for *in vitro* growth in normoxia as the cells derived their energy mainly from respiration (Fig. 6A, B and data not shown). These findings are in good agreement with the report of Wu and co-workers stating that A549 cells rely more on OXPHOS than on glycolysis [39]. However, phenformin, which induced substantially glycolysis (ECAR, Fig. 5A, B), did not rescue *in vitro* growth in normoxia (Fig. 6A). We believe that the low expression of MCT1 and MCT4, even in parental cells, precluded efficient glycolysis and therefore proliferation in normoxia. Another explanation could be related to the toxicity generated by an increase in reactive oxygen species (ROS) production as recently proposed by Shackelford and co-workers [32]. However, in hypoxia the three parental cell lines became insensitive to metformin/phenformin alone or in the presence of MCT1 inhibitor because MCT4 was highly expressed, thereby restoring glycolysis. By contrast, the BSG-null cells, and particularly in the presence of iMCT1, showed a high level of sensitivity to phenformin, which is consistent with ATP crisis or “metabolic catastrophe”, as discussed recently [8] and reported for colon adenocarcinoma and glioblastoma cells [19] (Fig. 6A, B).

Only the BSG-null tumours were sensitive to phenformin (Fig. 7). The fact that parental cells were totally insensitive to phenformin, which contrasted with the *in vitro* data, may suggest that the tumour cells were exposed to a hypoxic microenvironment favouring glycolysis and /or production of a low level of ROS. Another explanation for the attenuated sensitivity to phenformin *in vivo* in both parental and BSG-null cells is that the concentration of phenformin delivered *in vivo* was lower than the concentration of 50μM used *in vitro*.

Moreover, Shackelford and co-workers showed that inactivation of the suppressor gene *LKB1* dictated a therapeutic response of NSCLC to phenformin [6, 32]. In this context, they showed that cells with mutated LKB1 (as in A549 cells) were more sensitive to phenformin and that this effect was dependent on AMPK activation. They hypothesized that without phosphorylation of AMPK through LKB1, these cells lacked the mitophagy induced by the protein ULK1, a downstream target of AMPK. Thus cells accumulated defective mitochondria and did not neutralize ROS that induced apoptosis. This is an interesting explanation, however our studies contradict this explanation since we found a high sensitivity to phenformin independently of the LKB1 status. We propose instead that the level of sensitivity to phenformin is dictated by the glycolytic rate. In normoxia, the cell line with the lowest glycolytic rate (A549) was the most sensitive. A decrease in glycolysis by BSG disruption rendered the three cell lines equally sensitive to phenformin in normoxia and hypoxia.

*In vitro* studies of cells cultured in monolayers have given information about cell-autonomous metabolism, however they do not reflect the real physiological microenvironment in tumours. *In vivo* studies showed that BSG disruption did not reduce tumour growth as shown by other groups [4, 33]. In fact, tumour growth increased approximately 4-fold for *BSG^−/−^* cells compared to wt cells. These results could reflect a clonal growth effect. However, *in vitro* studies showed that these KO cells had the same proliferation rate as wt cells. In addition we observed that phenformin drastically inhibited tumour growth, with a stronger impact on *BSG^−/−^* cells compared to wt cells.

## CONCLUSION

The results obtained by inhibiting the MCTs/BSG complex are promising. Nevertheless, we do not yet assume that MCTs are effective targets for cancer therapy. More effort is needed to prove that inhibition of metabolism, more specifically lactate transport, may be an alternative therapeutic strategy to use in the treatment of certain types of cancer. Therefore, the present work is an attempt to provide new data supporting the exploitation of MCTs/BSG as targets in lung cancer therapy.

## MATERIALS AND METHODS

### Tissue samples

Representative formalin-fixed paraffin-embedded samples from primary lung tumour tissues were retrieved from the archives of the Department of Pathology of Hospital S. João, Porto, Portugal. The tumours were classified according to the WHO criteria [36]. This cohort included 50 cases of non-small cell lung cancer (NSCLC), 8 were squamous cell carcinomas (SCC), 29 adenocarcinomas (AC) and 13 from other types. Adjacent non-tumour tissue from each case was also selected.

### Cells and culture conditions

The lung adenocarcinoma cell lines A549 (obtained from American Type Culture Collection, Manassas, VA, USA) and H1975 (kindly provided by Dr. Patrick Brest, Fr) were maintained in Dulbecco's modified eagle medium (DMEM, Gibco, Life Technologies Corporation) supplemented with 10% foetal calf serum (FCS – Gibco Life Technologies Corporation), penicillin (10units/mL) and streptomycin (10μg/mL), in a humidified atmosphere of 5% CO_2_ at 37 °C. Incubation in hypoxia was carried out at 1% oxygen.

### Immunohistochemistry

Sections of 3-μm were used for immunohistochemical (IHC) analysis. IHC for MCT1 and CD147/BASIGIN was performed according to the avidin-biotin-peroxidase methodology (R.T.U. Vectastin Elite ABC kit; Vector Laboratories Inc.), as previously described by our group [26]. MCT4 IHC was performed with the Ultravision Detection System Anti-polyvalent, HRP (Lab Vision Corporation, Thermo Fisher Scientific), as previously described [21, 28]. In brief, paraffin embedded sections were deparaffinized in xylene and hydrated in a graded series of ethanol solutions. For antigen retrieval, slides were incubated either with 1mM EDTA buffered solution, pH 8.0 (CD147) or citrate buffer, pH=6.0 (MCT1, MCT4) for 20 min in a water bath at 98°C. After endogenous peroxidase inactivation, incubation with the primary antibody was performed overnight for MCT1 and CD147 and for 2 h for MCT4 at room temperature. Tissues were stained with 3,3′-diamino-benzidine (DAB+ Substrate System, DakoCytomation, Dako, Agilent Technologies Company) and counterstained with haematoxylin. Colon carcinoma tissue was used as a positive control for MCT1, MCT4 and CD147. Stained slides were evaluated and then photographed under a bright field microscope. Sections were scored for plasma membrane expression following a semi-quantitative criterion. The score used was the sum of the percentage of positive cells (0, negative; 1, less than 5% positive cells; 2, 25% to 50% positive cells; and 3, more than 50% positive cells) and the staining intensity (0, negative; 1, weak; 2, moderate; 3, strong). Scores between 0 to 3 were classified as negative and 4 to 6 as positive [26].

### Generation of *BSG*-null cells using Zinc Finger Nucleases (ZFNs)

To generate A549, H1975 and H292 KO cells for *BASIGIN*, two ZFN plasmids (designed by Sigma-Aldrich CKOZFN1227-1KT, CompoZr Custom ZFN) targeting *BASIGIN* exon 2 were transfected using JetPRIME® Transfection Reagent (Polyplus-transfection SA), according to the manufacturer's instructions. Transfected cells were detected by fluorescence-activated cell sorting (FACS), with a CD147 (MAB972, R&D Systems) primary antibody and with a PE-conjugated anti-mouse IgG (115-115-164, Jackson ImmunoResearch) secondary antibody. Negative cells for BSG were selected, sorted and cloned by dilution in 96 wells. The absence of BSG expression was confirmed by immunoblotting and only clones lacking the two alleles of *BSG* were analysed.

### [^14^C]-lactate uptake

[^14^C]-lactate uptake was measured to determine lactate transport in A549 cells, as previously published [17]. The protein concentration was determined to normalize the radioactivity to the protein content of the cells in each dish. [^14^C]-L-lactate uptake was expressed as counts per million per mg of protein.

### Intracellular lactate assay

A549 and H1975 cells (3×10^5^) were seeded in 12-well plates and allowed to adhere overnight. After incubation in hypoxia (1% O_2_) for 24h, cells were washed twice and maintained in DMEM without glucose, pyruvate and serum for 1h with either DMSO or iMCT1/2 (300nM). DMEM 5% FCS with either DMSO or iMCT1/2 was added and cells were collected at the indicated times (0, 1, 3 and 6h). For determination of the intracellular lactate concentration, cells were washed once with cold PBS1x and cold water and lysed with 200μL cold water. 50μL of cell lysate was assayed in 96-well plates in triplicate to measure the lactate concentration using a lactate colorimetric assay kit (K607-100, Biovision Incorporated).

### Immunoblotting

Cells were lysed in 1.5x SDS sample buffer and incubated for 15min at 95°C. Protein concentrations were determined using the BCA Assay. 40μg of protein was separated on 8% SDS polyacrylamide gels and transferred onto polyvinylidene difluoride membranes (Millipore). Blots were blocked in 5% non-fat milk in TN buffer (50 mM Tris-HCl pH7.4, 150 mM NaCl) and incubated overnight with the primary antibodies for CD147/BSG (1:500; MAB972, R&D Systems), MCT4 (1:1000; SC-50329, Santa Cruz Biotechnology) and for MCT1 (1: 3000; rabbit polyclonal antibodies against the C-terminal last 15 residues, prepared in the laboratory). The polyclonal antibody to arrest-defective-1 protein (ARD1) was used as loading control (1:30000). Bands were detected with the ECL system (Amersham Biosciences) after incubation of blots with secondary anti-mouse or anti-rabbit antibodies (Promega) coupled to horseradish peroxidase.

### Clonogenicity assay

A549 and H1975 wt and *BSG^−/−^* cells (2×10^3^) were seeded in 60-mm dishes and incubated for 24h to adhere. Medium was replaced with DMEM 10% FCS with and without iMCT1/2 AR-C155858 (300nM) or Phenformin (50M) or both for 10 days in normoxia and 15 days in hypoxia (1% O_2_). Dishes were stained with 5% Giemsa (Fluka) for 45min to visualize colonies.

### Metabolic analysis

The extracellular acidification rate (ECAR) and the oxygen consumption rate (OCR) were measured with a Seahorse XF analyser (Seahorse Bioscience, MA, USA). A549 cells (8×10^4^) and H1975 cells (1×10^5^) were seeded on seahorse plates and allowed to grow for 24h in normoxia. Prior to measurement, Plates were incubated for 45min in a non-CO_2_ incubator at 37°C with seahorse medium without glucose, pyruvate, serum or bicarbonate.

10mM of glucose (Sigma), 300nM of iMCT1/2 (AstraZeneca, UK), 50μM of phenformin (Sigma) and 1μM of oligomycin (Sigma) were injected. After each addition, three data points of 3min were undertaken to determine the oxygen and proton concentrations in the medium. The protein concentration was determined to normalize OCR and ECAR values.

### Tumour xenografts

Immunodeficient female nude mice with approximately 22g body weight were housed in groups of five, under specific pathogen-free, controlled ambient conditions. Animal studies were conducted according to Centre National de la Recherche Scientifique institutional guidelines (Ciepal n° NCE/-165). A549 wt and *BSG^−/−^* cells (2×10^6^/mouse) suspended in 300μL of serum-free DMEM supplemented with insulin-transferrin-selenium (Gibco, Life Technologies Corporation) were sub-cutaneously injected into the backs of the mice. Water containing 5% sucrose with and without 200mg/Kg phenformin (Sigma Aldrich) was added to the drinking water, and the daily intake fluids were monitored. Sucrose was added to make the drinking water palatable (MVCL Appleyard et al. 2012). Fresh phenformin was administered every two days. Tumours were measured every 2–3 days using a calliper and the volume was determined by using the formula: (4π/3) × L/2 × W/2 × H/2, where L represents length, W the width, and H the height. Tumours were harvested at approximately 1 cm^3^, fixed and paraffin-embedded.

### Statistics

For *in vitro* studies, the GraphPad prism 5 software was used, with the Student's *t*-test, considering significant values to be *p<0.05.*
